# Supplementary material for: An open-source framework for large-scale, flexible evaluation of biomedical text mining systems
Source: J Biomed Discov Collab. 2008 Jan 29;3:1. doi: 10.1186/1747-5333-3-1 (PMC2276192; doi:10.1186/1747-5333-3-1)
Supplement: Additional file 1 — Evaluation platform source code. The additional file contains the software evaluation framework discussed in this paper. The most current release can be downloaded from SourceForge.net [1]. See the accompanying README file for instructions on installation and use. Also included in the distribution is a collection of UIMA wrappers for some commonly used BioNLP tools and annotated corpora. [file 1747-5333-3-1-S1.zip › bionlp_uima_repository-1.0.1/docs/api/src-html/edu/uchsc/ccp/uima/cc/summary/comparison/AnnotationGroupID.html]

```
001    package edu.uchsc.ccp.uima.cc.summary.comparison;
002    
003    
004    public class AnnotationGroupID implements Comparable {
005        private String id;
006    
007        public AnnotationGroupID() {
008            this.id = "-1";
009        }
010    
011        public AnnotationGroupID(String id) {
012            this.id = id;
013        }
014    
015        public String getId() {
016            return id;
017        }
018    
019        public void setId(String id) {
020            this.id = id;
021        }
022    
023        @Override
024        public String toString() {
025            return this.id;
026        }
027    
028        public int compareTo(Object o) {
029            if (o instanceof AnnotationGroupID) {
030                AnnotationGroupID compareID = (AnnotationGroupID) o;
031                return compareID.getId().compareTo(this.id);
032            } else {
033                System.err.println("ERROR -- AnnotationGroupID: Cannot compare AnnotationGroupID to " + o.getClass().getName());
034                return -1;
035            }
036        }
037    
038        @Override
039        public boolean equals(Object obj) {
040            if (this.compareTo(obj) == 0) {
041                return true;
042            } else {
043                return false;
044            }
045        }
046    
047    //    public int compare(Object o1, Object o2) {
048    //        if (o1 instanceof AnnotationGroupID & o2 instanceof AnnotationGroupID) {
049    //            AnnotationGroupID id1 = (AnnotationGroupID) o1;
050    //            AnnotationGroupID id2 = (AnnotationGroupID) o2;
051    //            return id1.compareTo(id2);
052    //        } else {
053    //            System.err.println("ERROR -- AnnotationGroupID: Class in not an AnnotationGroupID: Class1=" + o1.getClass().getName() + "  Class2=" + o2.getClass().getName());
054    //            return -1;
055    //        }
056    //    }
057    
058        @Override
059        public int hashCode() {
060            return id.hashCode();
061        }
062        
063        
064        
065    }
```
